# Ionic Liquid-Promoted Synthesis of Novel Chromone-Pyrimidine Coupled Derivatives, Antimicrobial Analysis, Enzyme Assay, Docking Study and Toxicity Study

**DOI:** 10.3390/molecules23020440

**Published:** 2018-02-16

**Authors:** Shailee V. Tiwari, Julio A. Seijas, Maria Pilar Vazquez-Tato, Aniket P. Sarkate, Kshipra S. Karnik, Anna Pratima G. Nikalje

**Affiliations:** 1 Y.B. Chavan College of Pharmacy, Dr. Rafiq Zakaria Campus, Rauza Baug, Aurangabad, Maharashtra 431001, India; shailee2010@gmail.com; 2 Departamento de Química Orgánica, Facultad de Ciencias, Universidad of Santiago De Compostela, Alfonso X el Sabio, 27002 Lugo, Spain; julioa.seijas@usc.es (J.A.S.); pilar.vazquez.tato@usc.es (M.P.V.-T.); 3 Department of Chemical Technology, Dr. Babasaheb Ambedkar Marathwada University, Aurangabad, Maharashtra 431004, India; aniketpharma1@gmail.com (A.P.S.); karnikkshipra@yahoo.co.in (K.S.K.)

**Keywords:** ionic liquid, antifungal activity, antibacterial activity, molecular docking, cytotoxicity, *in vivo* acute oral toxicity

## Abstract

Herein, we report an environmentally friendly, rapid, and convenient ionic liquid ([Et_3_NH][HSO_4_])-promoted facile synthesis of ethyl 4-(6-substituted-4-oxo-4H-chromen-3-yl)-6-methyl-2-thioxo/oxo-1,2,3,4-tetrahydropyrimidine-5-carboxylate derivatives **4**(**a**–**f**) and 4-(6-substituted-4-oxo-4H-chromen-3-yl)-6-methyl-2-thioxo/oxo-1,2,3,4-tetrahydropyrimidine-5- carbohydrazide derivatives **6**(**a**–**f**). All the synthesized derivatives **4**(**a**–**f**) and **6**(**a**–**f**) were evaluated for their *in vitro* antifungal and antibacterial activity, by method recommended by National Committee for Clinical Laboratory Standards (NCCLS). The compound **6c** bearing a fluoro group on the chromone ring and oxygen and a hydrazino group (–NHNH_2_) on the pyrimidine ring, was found to be the most potent antibacterial compound amongst the synthesized derivatives. The compound **6f** bearing a methoxy group (–OCH_3_) on the chromone ring and sulphur group on the pyrimidine ring, was found to exhibit equipotent antifungal activity when compared with the standard drug miconazole. A d-alanine-d-alanine ligase (DdlB) enzyme assay study and an ergosterol extraction and quantitation assay study were performed to predict the mode of action of the synthesized compounds. A molecular docking study was performed to predict the binding interactions with receptors and mode of action of the synthesized derivatives. Further, analysis of the ADMET parameters for the synthesized compounds has shown that these compounds have good oral drug-like properties and can be developed as oral drug candidates. To establish the antimicrobial selectivity and safety, the most active compounds **6c** and **6f** were further tested for cytotoxicity against the human cancer cell line HeLa and were found to be non-cytotoxic in nature. An *in vivo* acute oral toxicity study was also performed for the most active compounds **6c** and **6f** and the results indicated that the compounds are non-toxic in nature.

## 1. Introduction

In the past few decades many drug-resistant human pathogenic microbes have been observed [1] and this is a serious public health dilemma in a wide range of infectious disease [2,3]. Failure in the antimicrobial treatment is mostly observed due to the resistance developed in the microbes, which leads to increased risks to mortality and sometimes contributes to complications. To prevail over this challenge the best way is the design and development of novel bioactive compounds which will be effective against strains which have developed resistance. In spite of the large number of antibiotics and chemotherapeutics available for medical use, antimicrobial resistance has created a substantial medical need for new classes of antimicrobial agents. The design and synthesis of novel antimicrobials agents will forever remain an area of gigantic connotation [4,5]. Novel and potent antimicrobial agents can be obtained by revamping the structure of the available antimicrobial agents or combining together two or more different active pharmacophores present in antimicrobial agents into one molecule.

Coumarins are an elite class of naturally occurring compounds with promising therapeutic perspectives [6,7]. This compound has become obligatory structural unit that is utilitarian in medicinal chemistry, with a wide variety of activities such as anticancer [8], antioxidant [9], antiplasmodial [10], antimalarial [11], antirhinovirus [12], antifungal [13] and antibacterial properties [14]. 4-oxo-4H-Chromen-3-carbaldehyde (3-formylchromone) is a most significant starting point for the synthesis of quite a lot of biologically active compounds due to the existence of an unsaturated keto functional group, a conjugated second carbonyl group at the C-3 position, and an electrophilic centre at the C-2 position. Much research has been focused on the inhibition of bacterial growth using naturally occurring coumarins such as xanthoxin, herniarin, umbelliferone, and scopoletin and on the antifungal activity of umbelliferone, scopoletin, and coumarin itself [15]. The coumarin scaffold is present in many antimicrobial agents such as semigrabrin, novobiocin, quercetin, apigenin, etc.

Nitrogen-containing heterocycles such as pyrimidine are a subject of continuous interest because of their diverse biological activities and occurrence in natural medicinal plants. Pyrimidine and its derivatives are used as pesticides, herbicides and insecticides [16,17]. Marketed antifungal drugs such as flucytosine, voriconazole and albaconazole also contain pyrimidine nuclei [18].

**Scheme 1:**
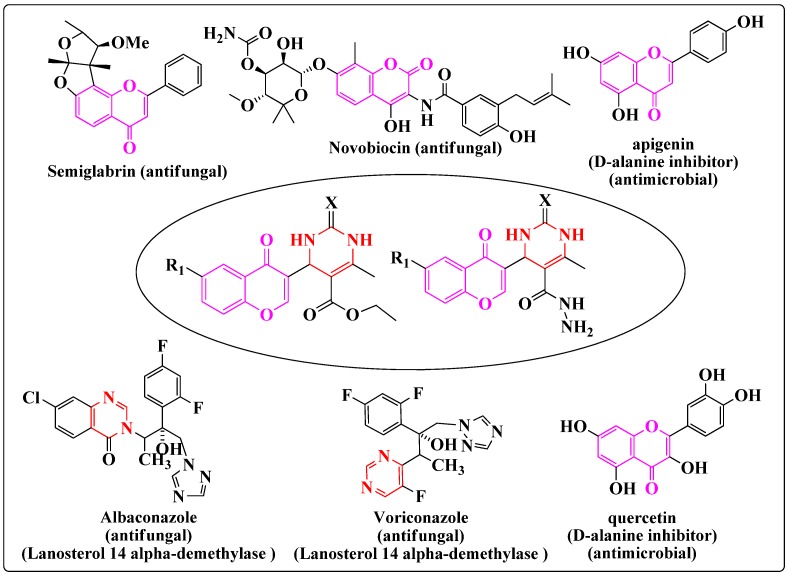
The design protocol for the synthesis of the target molecules.

Molecular hybridization is a rational drug design approach whereby new chemical entities are obtained by combining two or more pharmacophoric units from different bioactive compounds into a single molecule. Through this approach, medicinal chemists hope that the new hybrid derivatives will present better affinity and efficacy when compared to the individual parent drugs, a modified selectivity profile with improved pharmacokinetic and pharmacodynamic properties, dual or multiple modes of action, reduction of undesirable side effects, decreases in drug-drug interactions, reduced emergence or spread of drug resistance in microorganisms and protozoans and lower cost [19]. Pharmacophore hybridization is believed to be analogous to conventional combination therapy, with the exception that the two pharmacophores are covalently linked and available as a single entity. The selection of the two principles in the dual molecule is usually based on their observed (or anticipated) synergistic or additive pharmacological activities to enable the identification of highly active novel chemical entities [20,21,22,23]. In view of this and in continuation to our search for more effective antifungal and antibacterial agents [24,25,26,27,28], we planned the synthesis of coupled systems containing coumarin and pyrimidine rings with appropriate pharmacophores. The design protocol for the synthesized derivatives is as shown in Scheme 1. Considering the importance of both these scaffolds the molecular hybridization technique was used to couple them together, in the hope to getting newer derivatives with the higher antimicrobial activity.

Considering the focus on green synthesis in recent years, ionic liquids have attracted the attention of many researchers. Ionic liquids have been referred to as “designer solvents/green solvents” because of their physical and chemical properties which can be adjusted by varying the cation and anion. Taking into consideration the abovementioned points, we have carried out the synthesis of coumarin-pyrimidine coupled hybrid derivatives **4**(**a**–**f**) and **6**(**a**–**f**) using triethylammonium hydrogen sulphate [Et_3_NH][HSO_4_] as an solvent and easily recoverable green catalyst [26].

All the synthesized ethyl 4-(6-substituted-4-oxo-4H-chromen-3-yl)-6-methyl-2-thioxo/oxo-1,2,3,4-tetrahydropyrimidine-5-carboxylate derivatives **4**(**a**–**f**) and 4-(6-substituted-4-oxo-4*H*-chromen-3-yl)-6-methyl-2-thioxo/oxo-1,2,3,4-tetrahydropyrimidine-5-carbohydrazide derivatives **6**(**a**–**f**) were screened for their *in vitro* antifungal and antibacterial activity, by the method recommended by National Committee for Clinical Laboratory Standards (NCCLS), using miconazole and d-cycloserine as standard drugs, respectively. Minimum inhibitory concentration (MIC_100_) values were determined using the standard agar method as per CLSI guidelines [26,29,30,31].

The drug target selection was based on different biological roles such as: (i) cell wall synthesis; (ii) protein synthesis; (iii) nucleic acid synthesis; and (iv) cell metabolism [26]. ATP-dependent d-alanine-d-alanine ligase (Ddl) is one of the parts of biochemical machinery which is involved in biosynthesis of peptidoglycan, as it catalyzes the formation of the terminal d-ala-d-ala dipeptide of the peptidoglycan precursor UDP MurNAc pentapeptide. Bacterial growth is inhibited if the enzyme d-alanine ligase (Ddl) is been inhibited by the designed molecule. d-Alanine ligase (Ddl) enzyme inhibition is an attractive and tenable target in the search for novel and effective antimicrobial drugs [32]. Recently, coumarin derivatives have been reported to inhibit *E. coli* DdlB enzyme [33]. Hence, the designed and synthesized compounds were appraised for their inhibiton effect using d-alanine-d-alanine ligase (DdlB) enzyme assay study.

The main function of most antifungal agents is to prevent the synthesis of ergosterol, which is a key element of the fungal plasma membrane. Lanosterol 14α-demethylase enzyme plays an important role in ergosterol synthesis in fungi [18]. Exposure of fungi to lanosterol 14α-demethylase enzyme inhibitors causes reduction of ergosterol. This restricts and disturbs the structure of the membrane and its nutrient transport and chitin synthesis functions. The net result is to inhibit fungal growth. Most fungi cannot survive without ergosterol, and the enzymes that participate in its formation have become important targets for drug discovery. Recently, coumarin derivatives have been reported to inhibit cytochrome P450 lanosterol 14α-demethylase (CYP51) [34]. Based on literature reports [35] and the antifungal activity exhibited by the synthesized compounds, an ergosterol extraction and quantitation assay method was used to study the mode of action of the most potent antifungal compound **6f** [26].

Molecular docking is a well-known computational method for drug discovery which can be used to mock-up the interaction between a ligand and a protein at the atomic level and to predict, and characterize the behavior of ligands in the binding site of target proteins [36]. The synthesized compounds were thus docked into a homology model of cytochrome P450 lanosterol 14α-demethylase of *C. albicans* [37] to predict their antifungal activity mode of action. The synthesized compounds were also docked into the *E. coli* DdlB enzyme (PDB entry: 1IOV [38]) to predict its antibacterial activity mode of action [26].

To evaluate the drug-like properties of the synthesized compounds, the physicochemical parameters based on the Lipinski RO5 (Rule of Five) [39] and Jogersons rule of three were determined using Qikprop v. 3.5 (Schrodinger LLC). This pharmacological investigation of the compounds can support pharmaceutical scientist in selecting the most effective compounds for the development of better drugs [26]. Cytotoxicity studies and *in vivo* acute oral toxicity examinations of the most active synthesized compounds **6c** and **6f** were also performed.

## 2. Result and Discussion

### 2.1. Chemistry

**Scheme 2:**
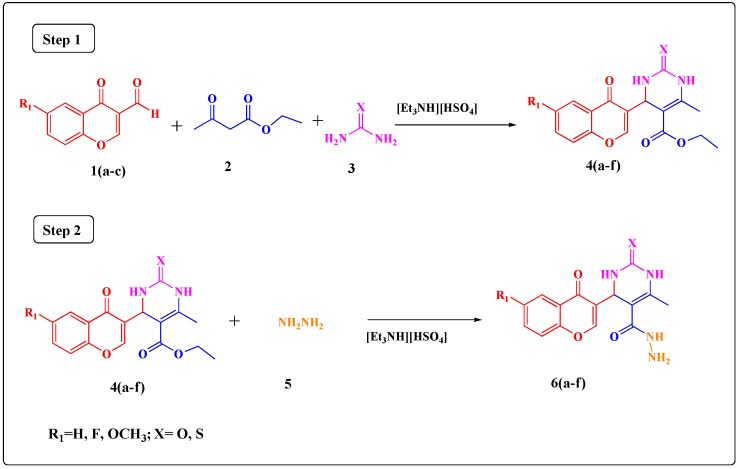
Scheme for green synthesis of the target compounds **4**(**a**–**f**) and **6**(**a**–**f**).

The utility of triethylammonium hydrogen sulphate [Et_3_NH][HSO_4_] in molten state, which is a low cost, mild, non-volatile and non-corrosive acidic ionic liquid, as an efficient Bronsted acid catalyst under solvent-free conditions for the Biginelli reaction is described, as shown in Scheme 2.

The model reaction of 4-oxo-4H-chromene-3-carbaldehydes, ethyl acetoacetate and urea in [Et_3_NH][HSO_4_] was optimized by investigating various parameters such as the mol percent of catalyst at various temperatures. The triethylammonium hydrogen sulphate [Et_3_NH][HSO_4_] ionic liquid was screened as catalyst at various loads such as 5, 10, 15, 20 mol % and at various temperatures such as 80, 90, 100, 110 °C as shown in Table 1. When 5 mol % of the catalyst was used at 100 °C the product **4a** was obtained in 72% yield in 150 min. Furthermore, the effect of the amount of catalyst was examined by examining the effect of various catalyst loads on the yield of product and the time required for completion of reaction for compound **4a**. When 10 mol % of catalyst was used at 100 °C, compound **4a** was obtained in 76% yield in 105 min. When 15 mol % of catalyst was used at 100 °C, 95% of **4a** was obtained in 60 min. The same yield resulted when 20 mol % of catalyst was used at 100 °C. Therefore, considering 15 mol % as an efficient amount the reaction was carried out at various temperatures like 80 °C, 90 °C, 100 °C and 110 °C. Temperatures both above and below 100 °C gave lower yields, therefore 15 mol % of the [Et_3_NH][HSO_4_] ionic liquid as catalyst and solvent at 100 °C was considered to give the best yield (95%) in the shortest reaction time (60 min) (Entry 3 of Table 1). 

These observations make the process under study more expeditious, economic, safe and eco-friendly. The recovery and reusability of the catalyst was investigated for the synthesis of compound **4a**. The results are given in Table 2; the recovered catalyst can be reused for at least four additional times in subsequent reactions without a considerable decrease in its catalytic activity.

A total of six novel ethyl 4-(6-substituted-4-oxo-4H-chromen-3-yl)-6-methyl-2-thioxo/oxo-1,2,3,4-tetrahydropyrimidine-5-carboxylate derivatives **4**(**a**–**f**) were synthesized according to the abovementioned green synthetic protocol. The time required for the completion of reactions was around 60–85 min time (monitored by TLC). The yields of the synthesized novel compounds were in the 86–95% range. The structures of the synthesized compounds were confirmed by IR, ^1^H-NMR, ^13^C-NMR, mass spectral analysis and elemental analysis.

The synthesized derivatives **4**(**a**–**f**) (1 mmol) were next allowed to react with hydrazine hydrate (1.2 mmol) under solvent free condition using [Et_3_NH][HSO_4_] as a catalyst and also as a solvent to furnish compounds **6**(**a**–**f**). The catalyst load and the temperature required for the synthesis of **6**(**a**–**f**) were also studied. The effect of different [Et_3_NH][HSO_4_] ionic liquid catalyst loads on the yield of product and time required for completion of reaction were studied at various catalyst loads such as 5, 10, 15 mol % and at various temperatures such as 70, 80, 90 and 100 °C, as shown in Table 3. When 5 mol % of the catalyst was used at 90 °C the product **6a** was obtained in 88% yield in 30 min. When 10 mol % catalyst was used at the same temperature, compound **6a** was obtained in 87% yield in 30 min. When 15 mol % catalyst was used at 90 °C, compound **6a** was obtained in 85% yield in 30 min. Therefore, considering 5 mol % as an efficient amount, the reaction was carried out at various temperatures like 70 °C, 80 °C, 90 °C and 100 °C. At 5 mol % catalyst loading the use of the higher (100 °C) or lower (70 °C, 80 °C) temperatures than 90 °C gave the compound **6a** in reduced yields after longer times, therefore, 5 mol % of the [Et_3_NH][HSO_4_] ionic liquid as catalyst and solvent was shown to give the best yield (88%) in a short reaction time (30 min) at 90 °C (Entry 3 of Table 3). The recovery and reusability of the catalyst in the synthesis of compound **6a** was also investigated. The findings are listed in Table 4; the recovered catalyst can be reused for at least four additional times in subsequent reactions without any considerable decrease in its catalytic activity.

A total of six 4-(6-substituted-4-oxo-4H-chromen-3-yl)-6-methyl-2-thioxo/oxo-1,2,3,4-tetra-hydropyrimidine-5-carbohydrazides 6(a–f) were synthesized according to the abovementioned green synthetic protocol. The time required for the completion of reactions was around 20–35 min (monitored by TLC), and the yields of the synthesized novel compounds were in the 80–95% range The structures of the synthesized compounds were confirmed by IR, 1H-NMR, 13C-NMR, mass spectral analysis and elemental analysis.

### 2.2. Biological Activity

#### 2.2.1. *In Vitro* Antibacterial Activity

All the synthesized compounds **4**(**a**–**f**) and **6**(**a**–**f**) were screened for their *in vitro* antibacterial activity against *Staphylococcus aureus (NCIM-2901), Escherichia coli* 1411 (standard laboratory strains) and *Escherichia coli* SM1411 (an *acrAB* deficient derivative of 1411). Minimum inhibitory concentration (MIC_100_) values were determined using methods recommended by National Committee for Clinical Laboratory Standards (NCCLS) [26,29,30,31]. Dimethyl sulfoxide (DMSO) was used as solvent control. The results of the *in vitro* antibacterial activity are presented in Table 5. The compound **6c** bearing a fluoro group on the chromone ring and oxygen and –NHNH_2_ groups on the pyrimidine ring was found to be the most potent antibacterial compound against *E. coli* 1411, *E. coli* SM1411 and *S. aureus*, with MIC_100_ values of 14 µg/mL, 14 µg/mL and 32 µg/mL, respectively. The compound **4c** was found to be the most potent antibacterial compound among the synthesized derivatives against *S. aureus*, with the MIC_100_ value 30 µg/mL.

The compound **6d** bearing a fluoro group on the chromone ring and –NHNH_2_ and sulphur groups on the pyrimidine ring was found to be equipotent to the standard drug d-cycloserine against *S. aureus,* with a MIC_100_ value of 32 µg/mL. The compounds **4d**, **4e**, **6e** and **6f** were found to be less active against *E. coli* 1411, *E. coli* SM1411 and *S. aureus.*

#### 2.2.2. *In Vitro* Antifungal Activity

The newly synthesized derivatives **4**(**a**–**f**) and **6**(**a**–**f**) were screened for their *in vitro* antifungal activity against different yeast and filamentous fungal pathogens. Minimum inhibitory concentration (MIC_100_) values for *in vitro* antifungal activity were determined by the methods recommended by National Committee for Clinical Laboratory Standards (NCCLS) [26,29,30,31]. Miconazole was used as standard drug. Dimethyl sulfoxide (DMSO) was used as solvent control. The MIC_100_ (µg/mL) of all the tested compounds and that of the reference drug miconazole are listed in Table 6. Results from Table 6 indicated that all the synthesized compounds showed good to moderate *in vitro* antifungal activity against the tested fungal strains.

The compounds **6f**, **6e** and **4e** were found to be the most potent antifungal agents among the synthesized derivatives. The compound **6f** bearing a –OCH_3_ group on the chromone ring and a sulphur group on the pyrimidine ring, was found to exhibit equipotent antifungal activity when compared with the standard drug miconazole. Compound **6f** had MIC_100_ values of 24 µg/mL for *C. albicans*, 25 µg/mL for *C. glabrata*, 28 µg/mL for *F. oxysporum*, 33 µg/mL for *A. fumigates*, 15 µg/mL for *A. flavus*, 16 µg/mL for *A. niger* and 15 µg/mL for *C. neoformans*. Compound **6e** was found to be the second most potent antifungal agent among the synthesized compounds, with MIC_100_ values 28 µg/mL for *C. albicans*, 30 µg/mL for *C. glabrata*, 30 µg/mL for *F. oxysporum*, 32 µg/mL for *A. fumigates*, 16 µg/mL for *A. flavus*, 18 µg/mL for *A. niger* and 16 µg/mL for *C. neoformans*. Compound **6f** exhibited more potent *in vitro* antifungal activity than the compounds **6b** and **6d**, which may be due to the presence of the electron donating –OCH_3_ substituent on the chromone ring. The compounds **4a**, **4b** and **4c** were found to be the least active antifungal agents among the synthesized series.

From the structure activity relationship (SAR) it could be manifested that the nuclei such as chromone and pyrimidine pharmacophores along with X = O/S are responsible for the antimicrobial activity. It could be deduced from the structure activity relationships (SARs), in general that the moderation on the chromone ring and the pyrimidine ring appreciably influenced the antimicrobial activity. The compounds **6c** with R_1_ = F group on the chromone ring and X = O group and –NHNH_2_ group on the pyrimidine ring was found to be more potent antibacterial agents than the standard drug d-cycloserine against all the tested bacterial strains. The compound **4c** with R_1_ = F group on the chromone ring and X = O group and –OC_2_H_5_ group on the pyrimidine ring was found to be a more potent antibacterial agent than the standard drug d-cycloserine against the *S. aureus* bacterial strain. The compound **6d** with R_1_ = F group on the chromone ring and X = S group and –NHNH_2_ group on the pyrimidine ring was found to be equipotent to the standard drug d-cycloserine against the *E. coli* bacterial strain. The compounds **4b**, **4e**, **4f**, **6b**, **6d** and **6f** with the sulphur group on the pyrimidine ring were found to be more moderately active antibacterial agents than those with oxygen groups on the pyrimidine ring **4a**, **4c**, **4d**, **6a**, **6c** and **6e**. Replacement of the –OC_2_H_5_ group with a –NHNH_2_ group on the pyrimidine ring significantly increased the *in vitro* antifungal activity. The compound **6f** with the –OCH_3_ group on the chromone ring and NHNH_2_ and S group on the pyrimidine ring was found to be the most active antifungal agent among the synthesized series against all the tested fungal strains. The compound **4e** (–OCH_3_ on the chromone ring and S group and –OC_2_H_5_ group on the pyrimidine ring) and compound **6e** (–OCH_3_ on the chromone ring and S group and on the pyrimidine ring) showed excellent *in vitro* antifungal activity against all the selected fungal strains. The compounds **4b**, **4e**, **4f**, **6b**, **6d** and **6f** with the sulphur group on the pyrimidine ring were found to be more active antifungal agents than those with oxygen group on the pyrimidine ring **4a**, **4c**, **4d**, **6a**, **6c** and **6e**. The chromone ring when substituted with an electron donating group such as R_1_ = OCH_3_ (**4d**, **4e**, **6e** and **6f**) was found to show better antifungal activity than when substituted with an electron withdrawing group like R_1_ = F (**4c**, **4f**, **6c** and **6d**).

#### 2.2.3. Ergosterol Extraction and Quantitation Assay

**Figure 1:**
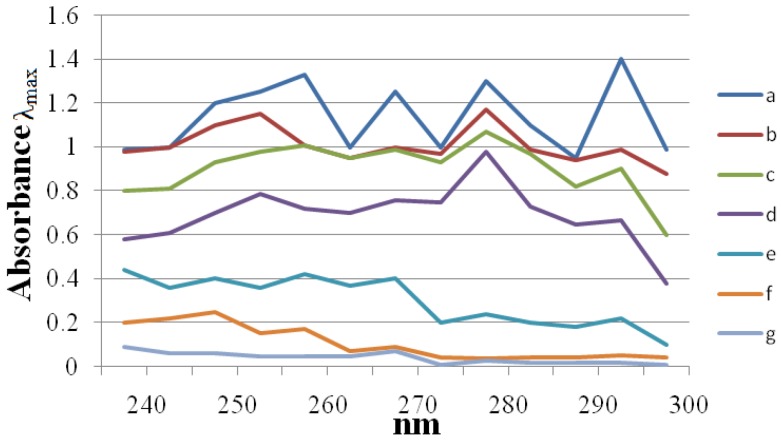
UV spectrophotometric sterol profile of *C. albicans* (NCIM3471) treated with, 0 (curve a), 1.5 (curve b), 3 (curve c), 6 (curve d), 12 (curve e) and 24 (curve f) µg/mL of synthesized compound **6f** and 25 (curve g) µg/mL of miconazole. Sterols were extracted from cells and spectral profiles between 240 and 300 nm were determined.

Considering that ergosterol is an important fungal cell membrane lipid, changes in its biosynthetic pathway may also cause damage to the fungal cell, preventing growth in a way similar as azole compounds such as miconazole, fluconazole, etc. do [36]. To reveal the antifungal mechanism of the most potent synthesized compound **6f**, its influence on the sterol composition on the *C. albicans* membrane was monitored by analyzing the changes in the sterol composition in the cells of *C. albicans* by U.V. analysis. The assay was performed at various concentrations of the most potent synthesized compound **6f** such as MIC/16, MIC/8, MIC/4, MIC/2 and MIC value to quantify the content of sterol produced by *C. albicans*, as shown in Figure 1.

Curve “a” in Figure 1 represents the negative control (no compound). The absorption of sterols extracted from fungal culture at the wavelengths of 230 nm, and 281.5 nm was analyzed and the results obtained are as shown in Figure 1 for compound **6f**. Ergosterol and an intermediate of the metabolic pathway, ergosterol-24(28) dehydroergosterol (DHE), absorb energy at 281.5 nm, but only DHE shows an intense absorption at 230 nm. Changes in this absorption pattern are therefore indicative of interference in the synthetic pathway of ergosterol [26,40]. There was a change in the absorption pattern for the synthesized compound **6f** as shown in Figure 1, which proves that the synthesized compound **6f** inhibits ergosterol biosynthesis by inhibiting the enzyme cytochrome P450 lanosterol 14 α-demethylase of *C. albicans*.

**Figure 2:**
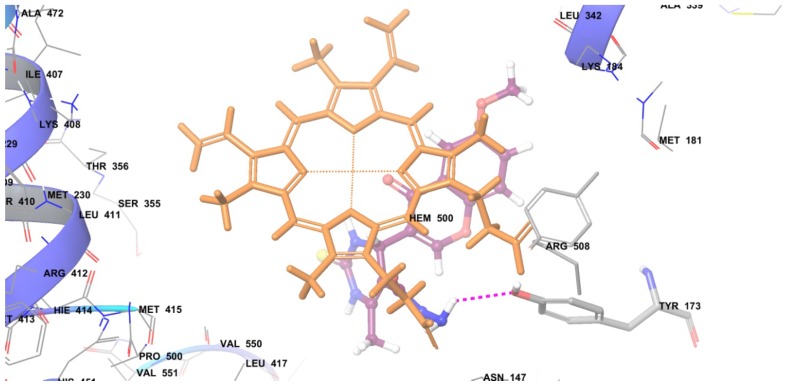
The docking pose of compound **6f** in the active pocket of cytochrome P450 lanosterol 14α-demethylase of *C. albicans.* (Light orange colored structure represents the heme co-*factor*, Pink bond represents the hydrogen bonding between ligand and receptor, Purple colored structure represents the molecule).

Curve “a” shows an intense ergosterol peak at 281.5 nm signifying the presence of this compound. Curve “g” in Figure 1 indicates the inhibition of ergosterol by miconazole (standard drug) at its MIC value. As the concentration of the synthesized compound **6f** increases the intensity of the ergosterol peak at 281.5 nm decreases, which indicates a decrease in the concentration of ergosterol in the culture medium. In Figure 2 there is almost a flat peak of compound **6f** at its MIC_100_ value, which is almost similar to that of standard drug miconazole at its MIC_100_ value. These results suggest that the compound **6f** might inhibit the important enzyme in fungus, i.e., lanosterol 14α-demethylase, in a similar manner to that accepted mechanism of the standard drug miconazole.

#### 2.2.4. d-Alanine-d-alanine ligase (Ddl) Enzyme Assay

The key enzyme involved in the peptidoglycan biosynthesis is d-alanine-d-alanine ligase (Ddl), making it an important target for drug discovery. Inhibition of DdlB as an essential enzyme in either Gram-positive or Gram-negative organisms leads to the loss of cell shape and integrity followed by bacterial death. The synthesized derivatives **4**(**a**–**f**) and **6**(**a**–**f**) were tested for their inhibitory activity on DdlB from *E.coli*. The results are presented as IC_50_ values. The compounds **4a**, **4c**, **6a**, **6b**, **6c** and **6d** were found to be better DdlB enzyme inhibitors than the standard drug d-cycloserine. The compound **6c** bearing R_1_ = fluoro on the chromone ring and X = O and –NHNH_2_ groups on the pyrimidine ring, was found to be the most potent DdlB enzyme inhibitor among the synthesized series. The compounds **4a**, **4c**, **4d**, **6a**, **6c** and **6e** with an oxygen group on the pyrimidine ring were found to be good inhibitors of DdlB enzyme.

### 2.3. Computational Study

#### 2.3.1. Molecular Docking

Docking is an effective and reliable approach to simulate the probable binding mode of ligands and proteins. Recently, coumarin derivatives have been reported to inhibit cytochrome P450 lanosterol 14α-demethylase (CYP51) [35]. Based on literature reports [36] and the antifungal activity exhibited by the synthesized compounds, molecular docking studies were performed in the active site of cytochrome P450 lanosterol 14α-demethylase enzyme of *C. albicans* [38] to understand its mechanism of action. *In silico* approaches like molecular docking have become very useful to identify potential targets for different ligands and they are associated with the thermodynamic interactions with the target enzyme governing the inhibition of the target microorganism [26]. The docking scores of the synthesized compounds **4**(**a**–**f**) and **6**(**a**–**f**) are presented in Table 7.

The docking data results revealed that the synthesized compounds were held in the active pocket by forming various interactions with the cytochrome P450 lanosterol 14α-demethylase of *C. albicans* such as hydrogen and hydrophobic interactions. The docking score of compound **6f** i.e −7.24 was found to be almost similar to that of standard drug miconazole i.e., −7.33. Figure 2 represents the docking pose of compound **6f** in the active pocket of cytochrome P450 lanosterol 14α-demethylase of *C. albicans* and it can be seen that the NH_2_ group of compound **6f** forms a hydrogen bond interaction with the HEM 500 and TYR 173. The phenyl ring containing a methoxy group fits well into the hydrophobic pocket of the enzyme.

On the basis of the *in vitro* antifungal activity, ergosterol extraction and quantitation assay and molecular docking study result, it was established that the synthesized compounds could inhibit the enzyme cytochrome P450 lanosterol 14α-demethylase of *C. albicans.* From the docking score data and the *in vitro* antifungal activity results it can be concluded that the compounds **4e**, **6e** and **6f** have good potential to inhibit the enzyme cytochrome P450 lanosterol 14α-demethylase of *C. albicans.*

Inhibition of Ddl in bacteria by drugs hinders the growth of bacteria, which makes this enzyme an irresistible and important target for the discovery of novel antimicrobial drug. Hence, molecular docking was performed against *E. coli* DdlB (PDB entry: 1IOV). The docking scores of the synthesized compounds **4**(**a**–**f**) and **6**(**a**–**f**) are presented in Table 8. The docking results indicated that the compounds were held in the active pocket by combination of various hydrogen and hydrophobic interactions with DdlB enzyme. The docking results revealed that, the highest binding compound to DdlB enzyme was compound **6c** with a G-Score of −6.71. 

**Figure 3:**
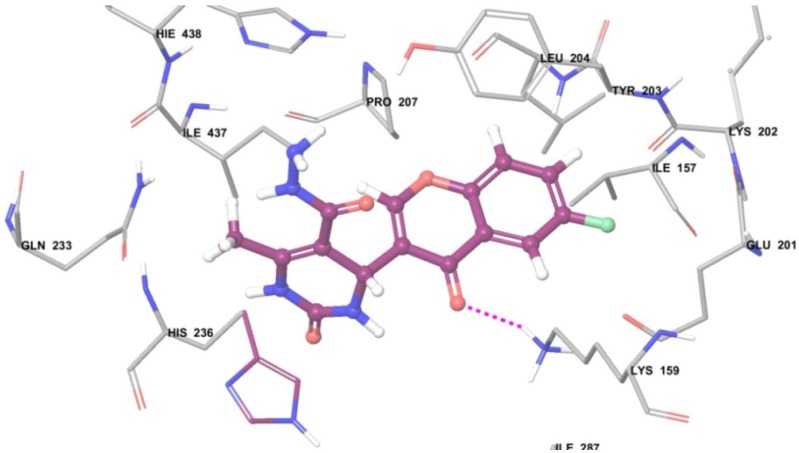
The docking pose of compound **6c** into the active pocket of DdlB enzyme. (Pink bond represents the hydrogen bonding between ligand and receptor, Purple colored structure represents the molecule).

From the *in vitro* antibacterial data, d-alanine-d-alanine enzyme assay study and molecular docking study it was found that the synthesized compounds **6a**, **6b**, **6c** and **6d** are capable of inhibiting the DdlB enzyme. Figure 3 represents the docking pose of compound **6c** into the active pocket of DdlB enzyme. The compound shows a hydrogen bond interaction with amino acid residue LYS159.

#### 2.3.2. *In Silico* Absorption, Distribution, Metabolism, Elimination and Toxicity (ADMET) Prediction

2D structures of all the synthesized ethyl 4-(6-substituted-4-oxo-4H-chromen-3-yl)- 6-methyl-2-thioxo/oxo-1,2,3,4-tetrahydropyrimidine-5-carboxylate and 4-(6-substituted-4-oxo-4H- chromen-3-yl)-6-methyl-2-thioxo/oxo-1,2,3,4-tetrahydropyrimidine-5-carbohydrazide derivatives **4**(**a**–**f**) and **6**(**a**–**f**) were subjected to *in silico* pharmacokinetic screening using Qikprop v. 3.5 (Schrodinger LLC) to study compliance with the Lipinski rule of five and Jorgensen’s rule of three. Understanding of *in silico* physicochemical pharmacokinetic parameters is imperative to predict the oral bioavailability of the synthesized compounds **4**(**a**–**f**) and **6**(**a**–**f**). It was contemplated that none of the synthesized compounds **4**(**a**–**f**) and **6**(**a**–**f**) violated Lipinski rule of 5 and Jorgensen’s rule of three, as shown in Table 9 and Table 10, respectively. The synthesized compounds **4**(**a**–**f**) and **6**(**a**–**f**) showed polar surface area (PSA) value ranges between 96.6 to 160.3 Å, as shown in Table 9, which suggests the good cell permeability properties of the synthesized compounds [41].

All the synthesized compounds **4**(**a**–**f**) and **6**(**a**–**f**) have Log S values within the desired range (−5.11–4.04) as shown in Table 10. The synthesized compounds showed BIP_Caco-2_ value ranges between 48.37 to 1137.55, which indicates good *in silico* intestinal absorption. The synthesized compounds exhibited MDCK values which range from 18.73 to 2820.05, indicating good *in silico* oral absorption [42]. The synthesized compounds showed Log Khsa values ranging between −0.01 to 0.24, as shown in Table 10, which is an indication that a significant proportion of the compounds are likely to circulate freely in the blood stream and hence reach the target sites. The most active antimicrobial compounds **6c, 6e** and **6f** showed 69.97%, 64.09% and 79.39% absorption, respectively. The HERG K^+^ channel, which is best known for its contribution to the electrical activity of the heart that coordinates the heart’s beating, appears to be the molecular target responsible for the cardiac toxicity of a wide range of therapeutic drugs [43]. Thus, HERG K^+^ channel blockers are potentially toxic and the predicted IC_50_ values often provide reasonable predictions for cardiac toxicity of drugs in the early stages of drug discovery [26,44]. From the Log HERG values in Table 10 it is observed that all the synthesized compounds **4**(**a**–**f**) and **6**(**a**–**f**) are non-toxic in nature.

### 2.4. In Vitro Cytotoxicity Study

**Figure 4:**
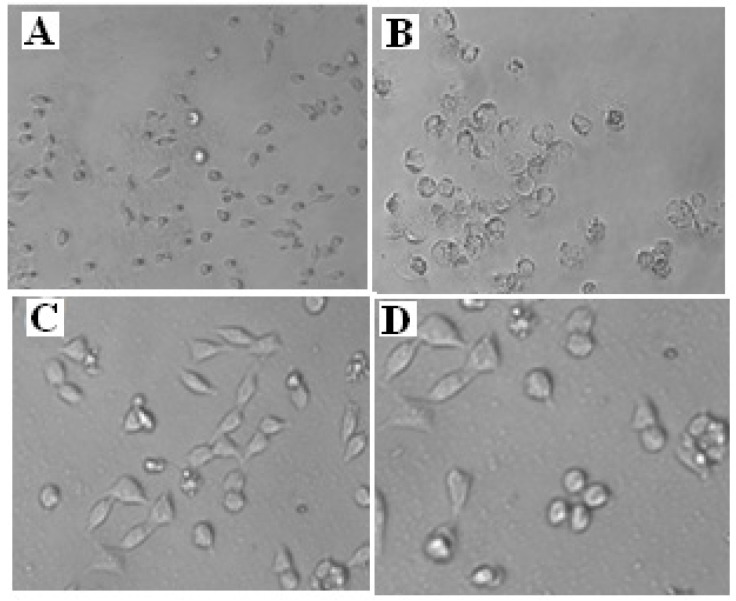
Images of *in vitro* cytotoxicity screening against HeLa (Human cervical cancer cell line) for Control (**A**), Positive control (**B**), Compound **6c** (**C**) at 80 µg/mL concentration, Compound **6f** (**D**) at 80 µg/mL concentration.

Toxicity scrutiny of synthesized compounds at the early phase of research paves the way to clinical trials and reduces the failure of potential therapeutics at later stages of clinical trials [24]. To scrutinize the safety and selectivity of antimicrobial activity of the synthesized compounds, the compounds **6c** and **6f** were appraised for their toxicity against HeLa (a human cervical cancer cell line) and PC-3 (human prostate cancer cell line) by the Sulforhodamine B (SRB) assay using adriamycin as a standard drug [26,45]. The cytotoxic effect of the synthesized compounds **6c** and **6f** was checked on cancer cell lines using the concentration range between 80 and 0.55 μg/mL, to determine the 50% growth inhibition (GI_50_). The results obtained are summarized in Table 11. The results indicate that in cytotoxicity studies, the most active synthesized compounds **6c** and **6f** can be considered as antimicrobial agent leads due to their lack of cell toxicity against HeLa at the maximum concentration evaluated. Figure 4 represents that cell inhibition did not occur even up to 80 µg/mL concentration of the synthesized compounds **6c** and **6f** and hence they can be considered as non-toxic in nature and selective in their antimicrobial activity.

### 2.5. In Vivo Acute Oral Toxicity Study and Behavioral Study

Animals treated with the newly synthesized compounds **6c** and **6f** were free of any toxicity as per acceptable range given by the OECD guideline No. 425 and no mortality was found up to 2000 mg/kg, which indicated that the lethal dose of the compounds is above 2000 mg/kg body weight in mice and that the compounds **6c** and **6f** can be considered to be safe and could be developed in the future as good antimicrobial agents. The *in vivo* acute oral toxicity and gross behavioral study results of the newly synthesized compounds **6c** and **6f** are as listed in Table 12 [26].

## 3. Materials and Methods

### 3.1. General Information

All the reactions were performed in oven-dried glassware. All reagents and solvents were used as obtained from the supplier or recrystallized/redistilled unless otherwise noted. The purity of the synthesized compounds was monitored by ascending thin layer chromatography (TLC) on silica gel-G (Merck, Darmstadt, Germany) coated aluminum plates, visualized by iodine vapor. Melting points were determined in open capillary tubes and are uncorrected. Infrared (IR) spectra were recorded on a PS 4000 FTIR instrument (JASCO, Tokyo, Japan) using KBr pellets. Elemental analyses (C, H, and N) were done with a FLASHEA 112 analyzer (Shimadzu, Mumbai, Maharashtra, India) and all analyses were consistent (within 0.4%) with theoretical values. The ^1^H-NMR and ^13^C-NMR spectra of synthesized compounds were recorded on an Avance II 400 NMR spectrometer (Bruker, Billerica, MA, USA) at 400/100 MHz frequency in DMSO-d_6_ or CDCl_3_ and using TMS as internal standard (chemical shift δ values are expressed in ppm). Mass spectra were scanned on a Micromass Q-Tof system (Waters, Manchester, UK) [26].

### 3.2. Synthesis of Ethyl 4-(6-Substituted-4-oxo-4H-chromen-3-yl)-6-methyl-2-thioxo/oxo-1,2,3,4-tetrahydro- pyrimidine-5-carboxylate Derivatives ***4**(**a**–**f**)*

A mixture of substituted 4-oxo-4*H*-chromene-3-carbaldehydes **1**(**a**–**c**) (1 mmol), ethyl acetoacetate (**2**) (1 mmol), urea/thiourea (**3**) (1 mmol) and [Et_3_NH][HSO_4_] (15 mol %) was refluxed under solvent-free conditions at 100 °C for the required time while the reactions were monitored by TLC. After completion of the reaction, the reaction mixture was poured into crushed ice and stirred for 5 min. The solid obtained was filtered, washed with cold water and then recrystallized from ethanol to afford the pure product. The ionic liquid present in the filtrate was recovered and was used for other reactions. 

*Ethyl 6-methyl-2-oxo-4-(4-oxo-4H-chromen-3-yl)-1,2,3,4-tetrahydropyrimidine-5-carboxylate* **4a**. Yield 95%; M.P.: 270–272 °C; IR (KBr *v*_max_ in cm^−1^): 3238 (N–H stretching), 3005 (C–H stretching), 2900 (–CH_3_ stretching), 2815 (alkyl CH stretching), 1746 (C=O stretching), 1601 (C=C stretching), 1454 (CH bending of CH_2_), 1356 (C–N stretching), 1002 (–O– stretching); ^1^H-NMR (DMSO-d_6_, δ_H_ ppm): 1.14 (t, *J* = 7.10 Hz, 3 H, CH_3_), 2.28 (s, 3 H, CH_3_), 4.05 (q, *J* = 7.04 Hz, 2 H, CH_2_), 4.72 (d, *J* = 1.49 Hz, 1H, CH), 7.41–7.59 (m, 3H, aromatic), 7.75 (s, 1H, NH), 8.05 (s, 1H, aromatic), 8.10 (d, *J* = 1.44 Hz, 1H, aromatic), 9.12 (s, 1H, NH); ^13^C-NMR (DMSO-d_6_, δ_C_ ppm): 14.45, 18.51, 45.91, 61.37, 101.94, 118.07, 121.61, 123.87, 126.27, 126.68, 133.85, 149.33, 150.72, 155.65, 155.77, 167.37, 172.17; MS: *m*/*z*: 329.21 [M + 1]^+^; Anal. Calcd. for C_17_H_16_N_2_O_5_: C, 62.19; H, 4.91; N, 8.53. Found: C, 62.20; H, 4.93; N, 8.50.

*Ethyl 6-methyl-4-(4-oxo-4H-chromen-3-yl)-2-thioxo-1,2,3,4-tetrahydropyrimidine-5-carboxylate* **4b**. Yield 87%; M.P.: 210–212 °C; IR (KBr *v*_max_ in cm^−1^): 3235 (N–H stretching), 3005 (C–H stretching), 2905 (–CH_3_ stretching), 2815 (CH alkyl stretching), 1600 (C=C stretching), 1454 (CH bending of CH_2_), 1360 (C–N stretching), 1140 (C=S stretching), 1002 (–O– stretching); ^1^H-NMR (DMSO-d_6_, δ_H_ ppm): 1.17 (t, *J* = 7.10 Hz, 3 H, CH_3_), 2.29 (s, 3 H, CH_3_), 4.74 (d, *J* = 1.49 Hz, 1H, CH), 7.47–7.74 (m, 3H, aromatic), 8.03 (s, 1H, NH), 8.06 (s, 1H, aromatic), 8.12 (d, *J* = 1.44 Hz, 1H, aromatic), 8.89 (s, 1H, NH); ^13^C-NMR (DMSO-d_6_, δ_C_ ppm): 14.50, 18.44, 50.62, 61.77, 106.24, 118.71, 121.32, 123.99, 126.12, 133.91, 150.11, 155.60, 160.39, 167.33, 171.94, 177.89; MS: *m*/*z*: 345.01 [M + 1]^+^; Anal. Calcd. for C_17_H_16_N_2_O_4_S: C, 59.29; H, 4.68; N, 8.13. Found: C, 59.31; H, 4.69; N, 8.10.

*Ethyl 4-(6-fluoro-4-oxo-4H-chromen-3-yl)-6-methyl-2-oxo-1,2,3,4-tetrahydropyrimidine-5-carboxylate* **4c**. Yield 92%; M.P.: 200–202 °C; IR (KBr *v*_max_ in cm^−1^): 3230 (N–H stretching), 3000 (C–H stretching), 2900 (–CH_3_ stretching), 2815 (CH alkyl stretching), 1745 (C=O stretching), 1600 (C=C stretching), 1454 (CH bending of CH_2_), 1364 (C–N stretching), 1002 (–O– stretching); ^1^H-NMR (DMSO-d_6_, δ_H_ ppm): 1.15 (t, *J* = 7.10 Hz, 3 H, CH_3_), 2.29 (s, *J* = 2.11 Hz, 3 H, CH_3_), 4.09 (q, *J* = 7.04 Hz, 2 H, CH_2_), 4.74 (d, *J* = 1.51 Hz, 1H, CH), 6.91–7.11 (m, 2 H, aromatic), 7.43 (s, 1 H, NH), 7.74 (d, *J* = 2.93 Hz, 1 H, aromatic) 8.06 (s, 1 H, aromatic), 9.13 (s, 1H, NH); ^13^C-NMR (DMSO-d_6_, δ_C_ ppm): 14.22, 18.11, 45.51, 61.70, 101.99, 111.52, 118.71, 119.23, 122.00, 126.88, 149.14, 150.79, 155.13, 156.11, 164.19, 167.19, 172.10; MS: *m*/*z*: 347.11 [M + 1]^+^; Anal. Calcd. for C_17_H_15_FN_2_O_5_: C, 58.96; H, 4.37; N, 8.09. Found: C, 58.99; H, 4.39; N, 8.04.

*Ethyl 4-(6-methoxy-4-oxo-4H-chromen-3-yl)-6-methyl-2-oxo-1,2,3,4-tetrahydropyrimidine-5-carboxylate* **4d**. Yield 88%; M.P.: 230–232 °C; IR (KBr *v*_max_ in cm^−1^): 3238 (N–H stretching), 3005 (C–H stretching), 2900 (–CH_3_ stretching), 2845 (–OCH_3_ stretching), 2815 (alkyl CH stretching), 1742 (C=O stretching), 1600 (C=C stretching), 1454 (CH bending of CH_2_), 1362 (C–N stretching), 1002 (–O– stretching); ^1^H-NMR (DMSO-d_6_, δ_H_ ppm): 1.17 (t, *J* = 7.10 Hz, 3 H, CH_3_), 2.29 (s, *J* = 2.11 Hz, 3 H, CH_3_), 3.80 (s, 3 H, O CH_3_), 4.10 (q, *J* = 7.04 Hz, 2 H, CH_2_) 4.74 (d, *J* = 1.51 Hz, 1H, CH), 7.37 (d, *J* = 8.90 Hz, 1H), 7.42 (s, 1H, NH), 7.48–7.52 (m, 2 H), 8.06 (s, 1H, aromatic), 9.13 (s, 1 H, NH); ^13^C-NMR (DMSO-d_6_, δ_C_ ppm): 14.45, 18.51, 45.91, 55.08, 61.37, 101.94, 107.44, 117.98, 119.35, 122.42, 149.33, 150.72, 152.63, 155.65, 156.18, 167.37, 171.94; MS: *m*/*z*: 359.53 [M + 1]^+^; Anal. Calcd. for C_18_H_18_N_2_O_6_: C, 60.33; H, 5.06; N, 7.82. Found: C, 60.35; H, 5.09; N, 7.80.

*Ethyl 4-(6-methoxy-4-oxo-4H-chromen-3-yl)-6-methyl-2-thioxo-1,2,3,4-tetrahydropyrimidine-5-carboxylate* **4e**. Yield 86%; M.P.: 222–224 °C; IR (KBr *v*_max_ in cm^−1^): 3235 (N–H stretching), 3008 (C–H stretching), 2908 (–CH_3_ stretching), 2845 (–OCH_3_ stretching), 2813 (alkyl CH stretching), 1740 (C=O stretching), 1600 (C=C stretching), 1454 (CH bending of CH_2_), 1360 (C–N stretching), 1148 (C=S stretching), 1002 (–O– stretching); ^1^H-NMR (DMSO-_d6, δH_ ppm): 1.17 (t, *J* = 7.10 Hz, 3 H, CH_3_), 2.29 (s, 3 H, CH_3_), 3.80 (s, 3 H, O CH_3_), 4.11 (q, *J* = 7.04 Hz, 2 H, CH_2_), 4.73 (d, *J* = 1.49 Hz, 1 H), 7.37 (d, *J* = 8.90 Hz, 1 H), 7.36 (s, 1 H, NH), 7.48–8.08 (m, 3 H), 8.89 (s, 1 H, NH); ^13^C-NMR (DMSO-d_6_, δ_C_ ppm): 14.45, 18.51, 49.08, 55.80, 61.37, 104.43, 107.44, 117.98, 119.35, 122.42, 126.68, 149.33, 152.63, 154.52, 156.18, 167.37, 171.94, 177.89; MS: *m*/*z*: 375.17 [M + 1]^+^; Anal. Calcd. for C_18_H_18_N_2_O_5_S: C, 57.74; H, 4.85; N, 7.48. Found: C, 57.77; H, 4.86; N, 7.46.

*Ethyl 4-(6-fluoro-4-oxo-4H-chromen-3-yl)-6-methyl-2-thioxo-1,2,3,4-tetrahydropyrimidine-5-carboxylate* **4f**. Yield 92%; M.P.: 228–230 °C; IR (KBr *v*_max_ in cm^−1^): 3230 (N–H stretching), 3000 (C–H stretching), 2910 (–CH_3_ stretching), 2810 (alkyl CH stretching), 1742 (C=O stretching), 1605 (C=C stretching), 1450 (CH bending of CH_2_), 1360 (C–N stretching), 1140 (C=S stretching), 1005 (–O– stretching); ^1^H-NMR (DMSO-d_6_, δ_H_ ppm): 1.15 (t, *J* = 7.10 Hz, 3 H, CH_3_), 2.29 (s, 3 H, CH_3_), 4.11 (q, *J* = 7.04 Hz, 2 H, CH_2_), 4.73 (d, *J* = 1.49 Hz, 1 H, CH), 6.91–7.74 (m, 3 H, aromatic), 7.76 (s, 1 H, NH), 8.08 (s, 1 H, aromatic), 8.89 (s, 1 H, NH); ^13^C-NMR (DMSO-d_6_, δ_C_ ppm): 14.44, 18.46, 50.66, 61.79, 106.33, 111.50, 118.73, 119.31, 122.18, 126.89, 149.15, 152.36, 160.19, 162.58, 167.79, 171.96, 177.88; MS: *m*/*z*: 363.15 [M + 1]^+^; Anal. Calcd. for C_17_H_15_FN_2_O_4_S: C, 56.35; H, 4.17; N, 7.73. Found: C, 56.37; H, 4.19; N, 7.70.

### 3.3. Synthesis of 4-(6-Substituted-4-oxo-4H-chromen-3-yl)-6-methyl-2-thioxo/oxo-1,2,3,4-tetrahydro- pyrimidine-5-carbohydrazide Derivatives ***6**(**a**–**f**)*

A mixture of ethyl4-(6-substituted-4-oxo-4*H*-chromen-3-yl)-6-methyl-2-thioxo/oxo-1,2,3,4-tetrahydropyrimidine-5-carboxylate derivative **4**(**a**–**f**) (1 mmol), hydrazine hydrate (**5**) (1.2 mmol) and [Et3NH][HSO4] (5 mol %) was refluxed under solvent-free conditions at 90 °C for the required time, while reaction completion was monitored by TLC. After completion of the reaction, the reaction mixture was poured into crushed ice and stirred for 5 min. The solid obtained was filtered, washed with cold water and then recrystallized from ethanol to afford the pure product. The ionic liquid present in the filtrate was recovered and was used for other reactions.

*6-Methyl-2-oxo-4-(4-oxo-4H-chromen-3-yl)-1,2,3,4-tetrahydropyrimidine-5-carbohydrazide* **6a**. Yield 88%; M.P.: 256–258 °C; IR (KBr *v*_max_ in cm^−1^): 3520 (NH_2_ stretching), 3450 (N–H stretching), 3000 (C–H stretching), 2900 (–CH_3_ stretching), 1742 (C=O stretching), 1605 (C=C stretching), 1368 (C–N stretching), 1005 (–O– stretching); ^1^H-NMR (DMSO-d_6_, δ_H_ ppm): 2.03 (s, 3H, CH_3_), 3.13 (s, 2 H, NH_2_), 4.74 (d, *J* = 1.51 Hz, 1H, CH), 7.08 (s, 1 H, NH), 7.42 (s, 1H, NH), 7.47–8.13 (m, 5H, aromatic), 9.39 (s, 1 H, NH); ^13^C-NMR (DMSO-d_6_, δ_C_ ppm): 18.49, 46.11, 102.31, 118.71, 121.61, 123.44, 126.23, 126.85, 133.88, 149.45, 150.71, 155.36, 159.17, 168.62, 172.49; MS: *m*/*z*: 315.25 [M + 1]^+^; Anal. Calcd. for C_15_H_14_N_4_O_4_: C, 57.32; H, 4.49; N, 17.83. Found: C, 57.32; H, 4.49; N, 17.83.

*6-Methyl-4-(4-oxo-4H-chromen-3-yl)-2-thioxo-1,2,3,4-tetrahydropyrimidine-5-carbohydrazide* **6b**. Yield 88%; M.P.: 198–200 °C; IR (KBr *v*_max_ in cm^−1^): 3515 (–NH_2_ stretching), 3450 (N–H stretching), 3000 (C–H stretching), 2908 (–CH_3_ stretching), 1742 (C=O stretching), 1600 (C=C stretching), 1360 (C–N stretching), 1145 (C=S stretching), 1002 (–O– stretching); ^1^H-NMR (DMSO-d_6_, δ_H_ ppm): 2.26 (s, 3 H, CH_3_), 3.13 (s, 2H, NH_2_), 4.74 (d, *J* = 1.49 Hz, 1 H, CH), 7.08 (s, 1 H, NH), 7.47 (s, 1 H, NH), 7.59–8.13 (m, 5 H, aromatic), 8.89 (s, 1H, NH); ^13^C-NMR (DMSO-d_6_, δ_C_ ppm): 18.97, 51.16, 106.11, 118.23, 121.14, 123.43, 126.28, 126.81, 133.69, 150.11, 155.49, 160.19, 168.66, 172.32, 177.80; MS: *m*/*z*: 331.29 [M + 1]^+^; Anal. Calcd. for C_15_H_14_N_4_O_3_S: C, 54.53; H, 4.27; N, 16.96. Found: C, 54.55; H, 4.29; N, 16.93.

*4-(6-Fluoro-4-oxo-4H-chromen-3-yl)-6-methyl-2-oxo-1,2,3,4-tetrahydropyrimidine-5-carbohydrazide* **6c**. Yield 95%; M.P.: 210–212 °C; IR (KBr *v*_max_ in cm^−1^): 3510 (NH_2_ stretching), 3455 (N–H stretching), 3000 (C–H stretching), 2910 (–CH_3_ stretching), 1742 (C=O stretching), 1605 (C=C stretching), 1365 (C–N stretching), 1005 (–O– stretching); ^1^H-NMR (DMSO-d_6_, δ_H_ ppm): 2.03 (s, 3 H, CH_3_), 3.13 (s, 2H, NH_2_), 4.74 (d, *J* = 1.51 Hz, 1 H, CH), 6.91 (d, *J* = 8.93 Hz, 1H), 7.08 (s, 1H, NH), 7.10–8.06 (m, 4 H, aromatic), 9.39 (s, 1H, NH); ^13^C-NMR (DMSO-d_6_, δ_C_ ppm): 18.26, 46.83, 106.12, 109.11, 118.36, 121.33, 123.39, 126.10, 147.77, 150.18, 155.60, 155.99, 162.45, 168.20, 172.45; MS: *m*/*z*: 333.55 [M + 1]^+^; Anal. Calcd. for C_15_H_13_FN_4_O_4_: C, 54.22; H, 3.94; N, 16.86. Found: C, 54.25; H, 3.96; N, 16.83.

*4-(6-Fluoro-4-oxo-4H-chromen-3-yl)-6-methyl-2-thioxo-1,2,3,4-tetrahydropyrimidine-5-carbohydrazide* **6d**. Yield 90%; M.P.: 146–148 °C; IR (KBr *v*_max_ in cm^−1^): 3512 (NH_2_ stretching), 3458 (N–H stretching), 3000 (C–H stretching), 2912 (–CH_3_ stretching), 1742 (C=O stretching), 1605 (C=C stretching), 1360 (C–N stretching), 1140 (C=S stretching), 1005 (–O– stretching); ^1^H-NMR (DMSO-d_6_, δ_H_ ppm): 2.27 (s, 3H, CH_3_), 3.14 (s, 2H, NH_2_), 4.74 (d, *J* = 1.49 Hz, 1H, CH), 6.92 (d, *J* = 8.93 Hz, 1H), 7.09 (s, 1H, NH), 7.11–8.09 (m, 4H, aromatic), 8.90 (s, 1 H, NH); ^13^C-NMR (DMSO-d_6_, δ_C_ ppm): 18.10, 52.61, 106.21, 109.14, 118.24, 121.39, 123.41, 126.52, 150.19, 155.62, 160.41, 162.56, 168.21, 171.96, 177.81; MS: *m*/*z*: 349.72 [M + 1]^+^; Anal. Calcd. for C_15_H_13_FN_4_O_3_S: C, 54.22; H, 3.94; N, 16.86. Found: C, 54.27; H, 3.99; N, 16.80.

*4-(6-Methoxy-4-oxo-4H-chromen-3-yl)-6-methyl-2-oxo-1,2,3,4-tetrahydropyrimidine-5-carbohydrazide* **6e**. Yield 82%; M.P.: 208–210 °C; IR (KBr *v*_max_ in cm^−1^): 3520 (NH_2_ stretching), 3452 (N–H stretching), 3000 (C–H stretching), 2905 (–CH_3_ stretching), 2845 (–OCH_3_ stretching), 1742 (C=O stretching), 1605 (C=C stretching), 1362 (C–N stretching), 1005 (–O– stretching); ^1^H-NMR (DMSO-d_6_, δ_H_ ppm): 2.03 (s, 3H, CH_3_), 3.13 (s, 2H, NH_2_), 3.80 (s, 3 H, OCH_3_), 4.74 (d, *J* = 1.51 Hz, 1 H), 7.08 (s, 1H, NH), 7.37–8.06 (m, 5 H, aromatic), 9.39 (s, 1H, NH); ^13^C-NMR (DMSO-d_6_, δ_C_ ppm): 18.92, 46.48, 56.82, 106.66, 109.11, 118.24, 121.48, 123.49, 125.76, 147.41, 150.62, 155.12, 156.30, 168.61, 172.27; MS: *m*/*z*: 345.02 [M + 1]^+^; Anal. Calcd. for C_16_H_16_N_4_O_5_: C, 55.81; H, 4.68; N, 16.27. Found: C, 55.84; H, 4.69; N, 16.25.

*4-(6-Methoxy-4-oxo-4H-chromen-3-yl)-6-methyl-2-thioxo-1,2,3,4-tetrahydropyrimidine-5-carbohydrazide* **6f**. Yield 80%; M.P.: 158–160 °C; IR (KBr *v*_max_ in cm^−1^): 3522 (NH_2_ stretching), 3455 (N–H stretching), 3000 (C–H stretching), 2910 (–CH_3_ stretching), 2845 (–OCH_3_ stretching), 1742 (C=O stretching), 1605 (C=C stretching), 1360 (C–N stretching), 1146 (C=S stretching), 1005 (–O– stretching); ^1^H-NMR (DMSO-d_6_, δ_H_ ppm): 2.26 (s, 3H, CH_3_), 3.13 (s, 2H, NH_2_), 3.80 (s, 3 H, OCH_3_), 4.74 (d, *J* = 1.49 Hz, 1 H), 7.08 (s, 1H, NH), 7.37–8.08 (m, 5H, aromatic), 8.89 (s, 1H, NH); ^13^C-NMR (DMSO-d_6_, δ_C_ ppm): 18.90, 51.50, 105.66, 106.41, 118.90, 121.71, 123.44, 126.47, 150.53, 150.66, 156.30, 160.31, 168.22, 171.96, 177.98; MS: *m*/*z*: 361.10 [M + 1]^+^; Anal. Calcd. for C_16_H_16_N_4_O_4_S: C, 53.32; H, 4.47; N, 15.55. Found: C, 53.35; H, 4.49; N, 15.51.

### 3.4. Biological Activity Assays

#### 3.4.1. *In Vitro* Antibacterial Activity

All the synthesized compounds **4**(**a**–**f**) and **6**(**a**–**f**) were screened for their *in vitro* antibacterial activity against *Staphylococcus aureus (NCIM-2901)* and *Escherichia coli* 1411 (standard laboratory strains) and *Escherichia coli* SM1411 (an *acrAB* deficient derivative of 1411). Minimum inhibitory concentration (MIC_100_) values were determined using the method recommended by National Committee for Clinical Laboratory Standards (NCCLS). *In vitro* antibacterial activities of the synthesized compounds **4**(**a**–**f**) and **6**(**a**–**f**) were tested in Nutrient Broth (NB) for Bacteria by the two fold serial dilution method. Seeded broth (broth containing microbial spores) was prepared in NB from 24 h old bacterial cultures on nutrient agar (Hi-media) at 37 ± 1 °C. The bacterial suspension was adjusted with sterile saline to a concentration of 1 × 10^−4^–10^−5^ C.F.U. The synthesized compounds and standard drug d-cycloserine were prepared by two fold serial dilutions to obtain the required concentrations. The tubes were incubated in BOD incubators at 37 ± 1 °C for bacteria. The MICs were recorded by visual observations after 24 h (for bacteria) of incubation [26,29,30,31].

#### 3.4.2. d-Alanine-d-alanine ligase (DdlB) Enzyme Inhibition Study

The d-Ala-adding activity of DdlB ligase was monitored by the detection of orthophosphate generated during the reaction based on the colorimetric malachite green method described by Walsh et al. [46]. Assays were performed at 37 °C in a mixture (final volume: 50 µL) containing 38.5 mM Hepes, pH 8.0, 3.25 mMMgCl_2_, 6.5 mM (NH_4_)_2_SO_4_, 700 µM d-Ala, 500 µM ATP, purified DdlB (diluted in 20 mM HEPES, pH 7.2, and 1 mM dithiothreitol) and the test compound (IC_50_ values were determined for a range of inhibitor concentrations). All the compounds were soluble in the assay mixture containing 5% DMSO. After 30 min of incubation, 100 µL Biomol reagent was added. After 5 min, absorbance was read at 650 nm. Residual activity was calculated with respect to a similar assay without inhibitor. To exclude possible non-specific (promiscuous) inhibitors, representative compounds **4**(**a**–**f**) and **6**(**a**–**f**) were also tested in the presence of Tween (0.003%), Triton X-114 (0.005%), and SDS (420 µM), as described by McGovern et al. [47,48]. No significant differences were found when compared to measurements without detergents [49].

#### 3.4.3. *In Vitro* Antifungal Activity

*In vitro* antifungal activity was determined as per CLSI (formerly, NCCLS) guidelines [29,30,31]. The synthesized compounds **4**(**a**–**f**)**, 6**(**a**–**f**) and the standard drug miconazole were dissolved in DMSO solvent. The medium yeast nitrogen base was dissolved in phosphate buffer pH 7 and it was autoclaved at 110 °C for 10 min. With each set a growth control without the antifungal agent and solvent control DMSO were included. The fungal strains were freshly sub cultured on to Sabouraud dextrose agar (SDA) and incubated at 25 °C for 72 h. The fungal cells were suspended in sterile distilled water and diluted to get 10^5^ cells/mL. Further, ten microlitres of the standardized suspension was inoculated onto the control plates and the media incorporated with the antifungal agents. The inoculated plates were incubated at 25 °C for 48 h. The readings were taken at the end of 48 h and 72 h. The MIC was the lowest concentration of drug preventing growth of macroscopically visible colonies on drug containing plates when there was visible growth on the drug free control plates [26].

#### 3.4.4. Ergosterol Extraction and Quantitation Assay

A single *Candida albicans* (NCIM-3471) colony from an overnight Sabouraud dextrose agar plate culture was used to inoculate 50 mL of Sabouraud dextrose broth for control and for various concentrations of the molecules. The cultures were incubated for 16 hours and harvested by centrifugation at 2700 rpm (856× *g*) for five min. The net weight of the cell pellet was determined. Three mL of 25% alcoholic potassium hydroxide solution was added to each pellet and vortex mixed for one min. Cell suspensions were transferred to sterile borosilicate glass screw-cap tubes and were incubated in an 85 °C water bath for one hour. Following incubation, the tubes were allowed to cool. Sterols were then extracted by addition of a mixture of 1 mL of sterile distilled water and 3 mL of *n*-heptane followed by vigorous vortex mixing for 3 min. The heptane layer was transferred to a clean borosilicate glass screw-cap tube and stored at −20 °C. Prior to analysis, 0.6 mL aliquot of sterol extract was diluted five fold in 100% ethanol and scanned spectrophotometrically between 240 nm and 300 nm with a spectrophotometer (UV-Visible Spectrophotometer 2100, Thermo Fischer Scientific, Waltham, MA, USA). The presence of ergosterol and the late sterol intermediate 24(28)-dehydroergosterol (DHE) in the extracted sample results in a characteristic four-peaked curve. The absence of detectable ergosterol in extracts was indicated by a flat line. A dose-dependent decrease in the height of the absorbance peaks was evident and corresponded to decreased ergosterol concentration [26,50].

### 3.5. Molecular Docking

The 3D model structure of cytochrome P450 lanosterol 14-α demethylase of *C. albicans* was built using homology modeling [37]. Molecular docking studies into a homology model of cytochrome P450 lanosterol 14 α-demethylase of *C. albicans* and into the *E. coli* DdlB enzyme (PDB entry: 1IOV [38]) were performed using Glide v. 6.8 (Schrodinger, LLC, New York, NY, USA, 2015) to predict its antifungal and antibacterial activity mode of action, respectively.

#### 3.5.1. Homology Modeling Procedure for Cytochrome P450 Lanosterol 14α-Demethylase of *C. albicans*

The 3D model structure of cytochrome P450 lanosterol 14α-demethylase of *C. albicans* was built using homology modeling. The amino acid sequence of the enzyme was obtained from the Universal Protein Resource (http://www.uniprot.org/) (Accession Code: P10613), and sequence homologous was obtained from Protein Data Bank (PDB) using Blast search. In literature, the structure of cytochrome P450 lanosterol 14α-demethylase was developed homologically using crystal structure of lanosterol 14α-demethylase from *C. albicans* as template. Based on the result of blast search, we used the crystal structure of human lanosterol 14-α DM (CYP51) with miconazole as a template for homology modeling (PDB ID. 3I3K). A hidden Markov model (HMM) was generated from a sequence alignment for the identification of sequence motifs and query family, which provides the information about which residues are conserved in the consensus sequence. This information was used as constraints in the generation of the protein sequence alignment. In addition, secondary structure prediction algorithms SSpro [51] were used for alignment of *C. albicans* lanosterol 14α-demethylase to human lanosterol 14α-demethylase (DM) enzyme. The combination of sequence motifs and secondary structure provides accurate picture of each helix. Alignment of *C. albicans* lanosterol 14α-demethylase with human lanosterol 14α-demethylase enzyme was carried out using the automated alignment program in PRIME v. 2.1 (Schrodinger LLC, New York, NY, USA). Following automated alignment, manual inspection was made to ensure the conserved motifs, and loops were correctly aligned. The final model of *C. albicans* lanosterol 14α-DM enzyme was developed using PRIME v2.1. The binding sites were generated using SITEMAP v2.3 [52] (Schrodinger LLC, New York, NY, USA), and side chain and loops around active binding site (site with highest sitemap score) were refined using Prime refinement tool. The quality of generated *C. albicans* lanosterol 14 α-demethylase model was assessed by using the well-validated programs PROCHECK [53] and WHATIF [37,54].

#### 3.5.2. Molecular Docking Study into *E. coli* DdlB Enzyme

Molecular docking study was performed in Maestro 9.1 using Glide v. 6.8 (Schrodinger LLC). All compounds were built using Maestro build panel and optimized to lower energy conformers using Ligprep v3.5 (Schrodinger, Inc., New York, NY, USA). The coordinates for *E. coli* DdlB enzyme were taken from RCSB Protein Data Bank and prepared for docking using ‘protein preparation wizard’ in Maestro v10.3. The bond orders and formal charges were added for hetero-groups and hydrogen’s were added to all atoms in the structure. Side chains that are not close to the binding cavity and do not participate in salt bridges were neutralized and termini were capped by adding ACE and NMA residue. After preparation, the structure was refined to optimize the hydrogen bond network using OPLS_2005 force field. The minimization was terminated when the energy converged or the RMSD reached a maximum cutoff of 0.30 Å. The extra precision (XP) docking mode for all compounds was performed on generated grid of protein structure [55]. The final evaluation of ligand-protein binding was done with Glide score [17].

### 3.6. In Silico ADMET Prediction

A computational study of the synthesized compounds **4**(**a**–**f**) and **6**(**a**–**f**) was performed for prediction of ADMET properties. The absorption, distribution, metabolism, elimination and toxicity (ADMET) properties of all the synthesized compounds were predicted using Qikprop v. 3.5 (Schrodinger LLC). In the present study, we have calculated the molecular volume (M.V.), molecular weight (M.W.), predicted octanol–water partition coefficient (log P o/w), number of hydrogen bond acceptors (*n*-ON), number of hydrogen bonds donors (*n*-OHNH), percentage human oral absorption (% ABS), Van der waals surface area of polar nitrogen and oxygen atoms (Polar Surface Area), Log S (water solubility), BIP_Caco-2_ (apparent Caco-2 cell permeability), Log Khsa (binding to human serum albumin) and Log HERG (toxicity study) [24].

### 3.7. In Vitro Cytotoxicity Studies

To explore the selective antifungal activity of the synthesized compounds *in vitro* cytotoxicity study of the most active compounds **6c** and **6f** was performed against HeLa (human cervical cancer cell line) and PC-3 (human prostate cancer cell line) using Sulforhodamine B (SRB) assay using Adriamycin as positive control [45]. The images were captured using an Eclipse Ti-S Inverted Research Microscope (Nikon, Tokyo, Japan). Image processing software used was NIS-Elements.

### 3.8. In Vivo Acute Oral Toxicity Study and Gross Behavioral Studies

Two of the newly synthesized compounds **6c** and **6f**, which are most potent among the synthesized compounds, were further subjected to an *in vivo* acute oral toxicity study and gross behavioral study as per the OECD guideline No. 425. Each group, consisting of six Swiss albino mice (18–22 gm weight, overnight fasted), were kept in colony cage at 25 ± 2 °C with 55% relative humidity and 12 h of light and dark cycle. A specified dose of 100, 250, 500, 750, 1000, 1500 and 2000 mg/kg body weight of mice was administered orally as a single dose. The acute toxic symptoms and the behavioral changes produced by the test compounds were observed continuously for 4 h periods at 8th, 12th and 24th h on set of toxic symptoms and the gross behavioral changes were also recorded. These animals were maintained for further 10 days with observation made daily. The *in vivo* acute oral toxicity study was carried out at animal house at the Y.B. Chavan College of Pharmacy, Aurangabad IAEC approval number CPCSEA/IAEC/P’col-52/2015-16/115 [26].

## 4. Conclusions

In this study, we have synthesized a suite of novel compounds **4**(**a**–**f**) and **6**(**a**–**f**) containing chromone-pyrimidine coupled hybrid derivatives as antifungal and antibacterial agents using a green synthetic protocol. The use of a green method, i.e., use of ionic liquid helped us in the synthesis of the target derivatives in good yield. The eco-friendly reaction conditions, good to excellent yields in a short reaction time and avoidance of cumbersome work-up procedures make this process economically worthwhile for industrial application, with the additional advantage of reusability of the catalyst. The *in vitro* antibacterial activity results suggest that compounds **4c**, **6c** and **6d** possess potent *in vitro* antibacterial activity. The *in vitro* antifungal activity results suggest that compounds **4e**, **6e** and **6f** possess potent *in vitro* antifungal activity. A d-alanine-d-alanine ligase (DdlB) enzyme assay study indicates that the synthesized derivatives have the ability to inhibit the DdlB enzyme. The synthesized derivative **6f** was found to be the most active antifungal agent; its mode of action was studied using an ergosterol extraction and quantitation assay method. The results of this test indicate that the synthesized compound **6f** acts by inhibiting ergosterol biosynthesis by inhibiting lanosterol 14α-demethylase enzyme. A molecular docking study was performed to predict the mechanism of action of the synthesized derivatives. Further, analysis of the ADMET parameters for the synthesized compounds performed using Qikprop v3.5 has revealed that these compounds have good oral drug-like properties and which makes them suitable for development as future oral drug candidates. Moreover, the cytotoxicity study revealed that the compounds **6c** and **6f** did not show any significant cytotoxicity against HeLa and PC-3 cell lines at the maximum evaluated concentration, thus indicating the selectivity of their antimicrobial action. The newly synthesized compounds **6c** and **6f** were also tested for their *in vivo* acute oral toxicity study and there was no mortality and no significant behavioral changes observed in Swiss albino mice for the first 24 h. Thus, the synthesized coupled compounds have shown excellent *in vitro* antimicrobial activity and compounds **4c**, **4e**, **6c** and **6f** could be in future further developed as lead antimicrobial molecules.

## Figures and Tables

**Table 1:** Effect of different reaction conditions on [Et_3_NH][HSO_4_] catalyzed synthesis of **4a**.

Entry	Temperature (°C)	Catalyst (mol %)	Time (min)	Yield (%)
1	100	5	150	77
2	100	10	105	76
3	100	15	60	95
4	100	20	60	95
5	110	15	65	91
6	90	15	80	70
7	80	15	85	70

**Table 2:** Reusability of [Et_3_NH][HSO_4_] catalyst for model reaction **4a**.

Entry	Run	Time	Yield
1	1	60	95
2	2	60	90
3	3	60	88
4	4	60	77

**Table 3:** Effect of different reaction conditions on [Et_3_NH][HSO_4_] catalyzed synthesis of **6a**.

Entry	Temperature (°C)	Catalyst (mol %)	Time (min)	Yield (%)
1	70	5	48	75
2	80	5	42	80
3	90	5	30	88
5	100	5	30	85
6	90	10	30	87
7	90	15	30	85

**Table 4:** Reusability of [Et_3_NH][HSO_4_] catalyst for model reaction **6a**.

Entry	Run	Time	Yield
1	1	30	88
2	2	30	86
3	3	30	85
4	4	30	80
5	5	30	72

**Table 5:** *In vitro* antibacterial activity of the synthesized compounds **4**(**a**–**f**) and **6**(**a**–**f**).

Compound	IC_50_ (µM)	MIC_100_^a^ (µg/mL)		
		*E. coli 1411*	*E. coli* SM1411	*S. aureus* NCIM-2901
**4a**	158	46	40	64
**4b**	296	50	50	68
**4c**	155	38	34	**30**
**4d**	330	55	54	>200
**4e**	335	58	>60	>200
**4f**	280	48	44	72
**6a**	127	34	32	66
**6b**	124	34	34	76
**6c**	**106**	**14**	**14**	**32**
**6d**	**111**	**16**	**18**	**40**
**6e**	315	>60	58	156
**6f**	>400	>60	>60	>200
d-Cycloserine	276	16	16	32

^a^ Values are the average of three readings; *E.coli 1411: Escherichia coli 1411, E.coli SM1411 Escherichia coli SM 1411; S. aureus NCIM-2901: Staphylococcus aureus (NCIM-2901)*; MIC: Minimum inhibitory concentration; Bold value indicates equipotent /nearness to values of standard drug d-Cycloserine.

**Table 6:** *In vitro* antifungal activity of the synthesized compounds **4**(**a**–**f**) and **6**(**a**–**f**).

Compound	MIC _100_^a^ (µg/mL)						
	*C. alb.*	*C. glab.*	*F. oxy.*	*A. fum.*	*A. fla.*	*A. nig.*	*C. neo.*
**4a**	>100	>100	82	65	66	57	60
**4b**	64	66	58	60	66	52	55
**4c**	52	58	58	62	58	44	46
**4d**	34	38	36	**36**	**20**	**20**	**24**
**4e**	**30**	**32**	**30**	**34**	**18**	**18**	**20**
**4f**	46	44	48	55	34	34	30
**6a**	78	68	76	60	68	55	58
**6b**	64	60	54	58	60	48	50
**6c**	52	52	58	60	42	40	40
**6d**	44	40	44	52	30	28	28
**6e**	**28**	**30**	**30**	**32**	**16**	**18**	**16**
**6f**	**24**	**25**	**28**	**33**	**15**	**16**	**15**
Miconazole	25	25	25	35	12	12	12

^a^ Values are the average of three readings; *C. alb.: Candida albicans; C. glab.: Candida glabrata; F. oxy.: Fusarium oxysporum; A. fum.: Aspergillus fumigates; A. nig.: Aspergillus niger; C. neo.: Cryptococcus neoformans*; MIC_100_: Minimum inhibitory concentration; Miconazole: Standard drug; Bold value indicates equipotent /nearness to values of standard drug Miconazole.

**Table 7:** The docking scores of the synthesized compounds **4**(**a**–**f**) and **6**(**a**–**f**) for cytochrome P450 lanosterol 14α-demethylase enzyme of *C. albicans*.

Compound	Docking Score	Compound	Docking Score
**4a**	−5.88	**6a**	−5.30
**4b**	−6.20	**6b**	−5.43
**4c**	−6.96	**6c**	−7.01
**4d**	−6.99	**6d**	−7.21
**4e**	−7.00	**6e**	−6.96
**4f**	−6.80	**6f**	−7.24
		Miconazole	−7.33

**Table 8:** Docking score of the synthesized compounds **4**(**a**–**f**) and **6**(**a**–**f**) for DdlB enzyme.

Compound	Docking Score	Compound	Docking Score
**4a**	−6.00	**6a**	−6.48
**4b**	−5.66	**6b**	−6.33
**4c**	−6.56	**6c**	−6.71
**4d**	−2.46	**6d**	−6.41
**4e**	−3.00	**6e**	−1.81
**4f**	−3.76	**6f**	−0.89
		d-Cycloserine	−6.22

**Table 9:** *In silico* physicochemical pharmacokinetic parameters important for good bioavailability according to Lipinski rule of 5 for the synthesized compounds 4(a–f) and 6(a–f).

Compound	M.W ^a^	Log P o/w ^b^ (−2.0 to 6.5)	*n*-ON ^c^ (<10)	*n*-OHNH ^d^ (<5)	PSA ^e^ (7–200.0)	Lipinski Rule of 5 (≤1)
**4a**	328.3	2.7	4.5	0	117.8	0
**4b**	344.3	3.4	5.0	0	96.6	0
**4c**	346.3	3.0	4.5	0	118.0	0
**4d**	358.3	2.8	5.2	0	125.9	0
**4e**	374.4	3.5	5.7	0	104.2	0
**4f**	362.3	3.6	5.0	0	97.2	0
**6a**	314.1	1.2	5.5	3	149.3	0
**6b**	330.3	1.9	6.0	3	128.5	0
**6c**	332.2	1.4	5.5	3	150.0	0
**6d**	348.3	2.0	6.0	3	130.4	0
**6e**	344.3	1.9	6.2	3	160.3	0
**6f**	360.3	1.9	6.7	3	137.5	0
Miconazole	402.1	6.6	5.0	1	67.1	1

^a^ Molecular weight of the molecule; ^b^ Predicted octanol–water partition coefficient (log Po/w) (−2.0 to 6.5); ^c^ *n*-ON number of hydrogen bond acceptors ≤10; ^d^ *n*-OHNH number of hydrogen bonds donors ≤5; ^e^ Polar surface area (PSA) (7.0–200.0); Miconazole: Standard drug.

**Table 10:** *In silico* physicochemical pharmacokinetic parameters important for good oral bioavailability according to Jorgensen’s rule of 3 for the synthesized compounds **4**(**a**–**f**) and **6**(**a**–**f**).

Compound	LogS ^a^ (−6 to 0.5)	BIP_Caco-2_^b^ (<25 Poor, >500 Good)	MDCK ^c^ (<25 Poor, >500 Good)	log Khsa ^d^ (−1.5 to 1.2)	% ABS ^e^	Log HERG ^f^ (Below −5)	Toxicity
**4a**	−4.33	271.70	120.96	0.24	86.50	−4.88	N
**4b**	−4.72	1032.02	1427.30	0.19	100	−4.94	N
**4c**	−4.92	254.65	203.96	0.31	87.66	−4.98	N
**4d**	−4.62	246.14	108.71	0.24	86.28	−4.98	N
**4e**	−5.00	1137.55	1583.83	0.19	100	−5.01	N
**4f**	−5.11	1121.26	2820.05	0.23	100	−4.90	N
**6a**	−3.81	86.72	35.20	−0.03	69.11	−4.75	N
**6b**	−4.39	265.39	328.59	−0.03	81.81	−5.04	N
**6c**	−3.98	82.41	60.29	0	69.97	−4.69	N
**6d**	−4.60	190.52	415.33	0	79.95	−4.91	N
**6e**	−4.04	48.37	18.73	−0.01	64.09	−4.83	N
**6f**	−4.58	195.06	235.15	−0.01	79.39	−4.97	N
Miconazole	−5.80	4747.2	10000	0.83	100	−5.5	N

^a^ Logarithm of Aqueous solubility (Log S) (−6 to 0.5); ^b^ Predicted apparent Caco-2 cell permeability, in nm/sec (BIP_Caco**-2**_) (<25 poor, >500 good); ^c^ Predicted apparent MDCK cell permeability in nm/sec (MDCK) (<25 poor, >500 good); ^d^ Logarithm of predicted binding constant to human serum albumin (log Khsa) (−1.5 to 1.2); ^e^ Percentage human oral absorption (% ABS) (>80% is high, <25% is poor); ^f^ Predicted IC_50_ value for blockage of HERG K^+^ channels (Log HERG) (below −5); N: Non-toxic.

**Table 11:** *In vitro* cytotoxicity study of the synthesized compounds **6c** and **6f**.

Compound	GI _50_ (µg/mL) ^a^	
	HeLa	PC-3
**6c**	>80	>80
**6f**	>80	>80
Adriamycin	0.5	0.5

^a^ GI_50_ is the concentration exhibiting 50% inhibition of the growth as compared to the growth of control; HeLa (human cervical cancer cell line); PC-3 (human prostate cancer cell line); adriamycin: standard drug.

**Table 12:** *In vivo* acute oral toxicity study and gross behavioral studies of the synthesized compounds **6c** and **6f**.

	Observations													
Entry	Toxicity	No. of Deaths	Eyes Lacrimation	Salivation	Diarrhea	Respiration Problem	Straub Tail	Pilo Erection	Convulsions	Motor Activity Normal	Tremors	Sedation	Hypnosis	Muscle Spasm
**6c**	N	N	N	N	N	N	N	Y	N	Y	N	N	N	N
**6f**	N	N	N	N	N	N	N	Y	N	Y	N	N	N	N

N: No, Y: Yes.
